# Not All Back Pain Is Muscle Strain: A Case of Epidural Abscess

**DOI:** 10.7759/cureus.42094

**Published:** 2023-07-18

**Authors:** Sheri P Walls, Olawole Akinboboye, Danhely Cruz, Tyler McMartin, Michell López Luciano

**Affiliations:** 1 Internal Medicine, Piedmont Athens Regional, Athens, USA; 2 Internal Medicine, Piedmont Athens regional, Athens, USA

**Keywords:** spinal abscess, abscess, bacaterial infection, infection, epidural abscess, lumbar abscess

## Abstract

Epidural abscesses are rare suppurative abscesses of the central nervous system that can expand and lead to severe neurologic complications and even death. Here we describe the case of a 68-year-old female who developed a spinal epidural abscess one month following cervical spinal decompression and fusion. The patient presented with decreased grip strength, flaccid paralysis of the lower extremities with hyporeflexia, urinary incontinence, and decreased sensation in the bilateral lower extremities. A cervical spine MRI revealed a large cervical spinal epidural abscess causing multilevel spinal cord compression that was treated with surgical evacuation and antibiotics. Due to the complications of epidural abscess, we as clinicians must have high clinical suspicion to initiate the correct treatment. In addition, patients without neurological symptoms or symptoms lasting less than 36 hours have the best recovery rate. Our case highlights the importance of timely diagnosis, management, and intervention, which can lead to restored functionality and the prevention of permanent neurologic sequelae.

## Introduction

An epidural abscess is a rapidly emerging infection that can be located within a patient's skull or spinal cord. Risks include a wide array of conditions such as immunosuppression (diabetes, HIV, malignancy, corticosteroid use, systemic infection), obesity, intravenous drug use, end-stage kidney disease, and end-stage liver disease [1]. The etiology of infections is mainly secondary to gram-positive organisms but may also be caused by gram-negative bacteria and, rarely, anaerobic bacteria and fungi [1]. Prevalence ranges from around 20 cases per 10,000 hospital admissions, and mortality increases if the diagnosis is not made timely; there could be severe complications [1]. The severity of complications is secondary to the location of the infection; it can lead to irreversible damage such as paralysis, kyphosis, osteomyelitis, endocarditis, and spinal compression. Epidural abscesses are rare suppurative abscesses of the central nervous system that can expand and lead to severe neurologic complications and even death. Here we describe the case of a 68-year-old female who developed a spinal epidural abscess one month following cervical spinal decompression and fusion. The patient presented with decreased grip strength, flaccid paralysis of the lower extremities with hyporeflexia, urinary incontinence, and decreased sensation in the bilateral lower extremities. A cervical spine MRI revealed a large cervical spinal epidural abscess causing multilevel spinal cord compression that was treated with surgical evacuation and antibiotics. Our case highlights the importance of timely diagnosis and intervention, which can lead to restored functionality and the prevention of permanent neurologic sequelae.

Epidural abscesses are rare abscesses of the central nervous system that require prompt diagnosis and treatment, given their ability to expand and compress the brain and spinal cord. Although rare, the incidence of epidural abscesses appears to be rising with the increase of spinal surgeries, IV catheters, and immunosuppression [2]. Even more rare are abscesses after cervical spine surgery. Epidural abscesses usually present acutely, and symptoms include back pain, paresthesias, fever, and neurological symptoms [3].

## Case presentation

**Figure 1:**
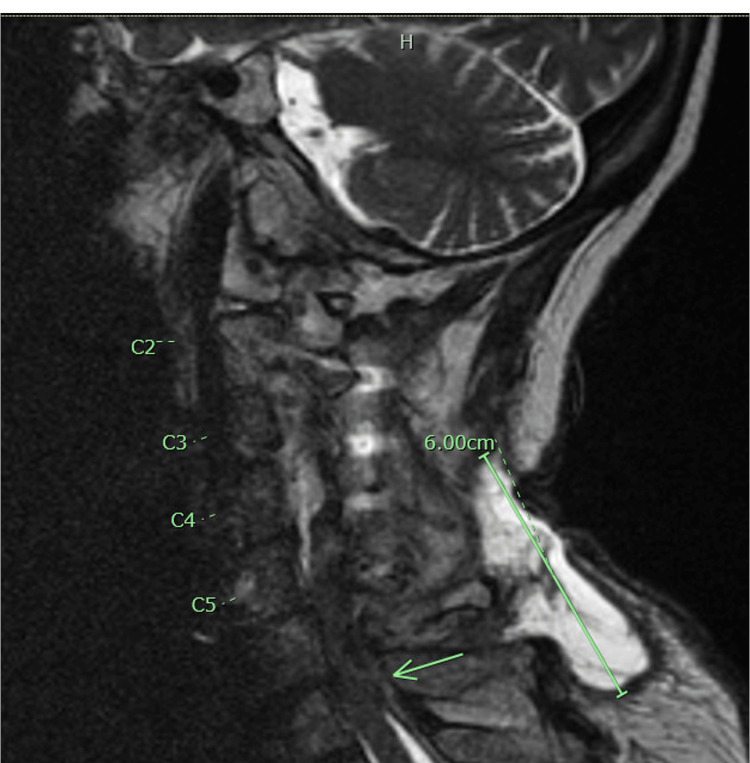
Epidural abscess

A 68-year-old female with a significant past medical history of chronic neck pain status post-recent L4-L5 spinal decompression and fusion, peripheral neuropathy, bipolar disorder, and type 2 diabetes presented to the emergency department for evaluation of weakness and surgical site pain. The patient underwent cervical spine surgery one month prior and had progressively worsening weakness that was associated with worsening ambulation. At the time of the initial encounter, the patient had 5/5 strength in the upper extremities, decreased grip strength, 0/5 lower extremity weakness with hyporeflexia, urinary incontinence, decreased sensation bilaterally in the lower extremities, and loss of perianal sensation. Patients’ initial findings were suggestive of cauda equina syndrome due to loss of perianal sensation, but only a multilevel disc bulge with spinal stenosis at L2-L3 on initial CT lumbar spine imaging was seen. The CBC and CMP were unremarkable. Due to persistent symptoms and concerns of flaccid paralysis and possible localization of the C7-T1 process due to the patient's neurological symptoms. A cervical spine MRI was ordered and a neurosurgical consultation was scheduled. MRI revealed, as noted in Figure 1, C5-C7 stenosis and cord compression secondary to epidural abscess, as well as T10-T11 stenosis for which emergent evacuation by surgical intervention was performed. The patient underwent C4-T1 posterior cervical decompression and evacuation of an epidural abscess. C5-T2 fusion and placed on post-operative drainage. 

Infectious disease was consulted for antibiotic management and recommended vancomycin and ceftriaxone for a total of six weeks secondary to MRSA that was found on culture. The patient gradually regained her grip and lower extremity strength. The patient was evaluated by physical therapy and placed in a rehab facility. 

## Discussion

As previously mentioned, epidural abscesses are rare infections that can be difficult to diagnose and eventually cause significant morbidity. It is important to have a high index of suspicion, as these can go untreated, putting the patient's life at risk. Patients with immunocompromised status, IVDU, recent surgery, and bacteremia have the highest risk for epidural abscesses. Most patients become infected through hematogenous dissemination, with skin abscesses and furuncles being the most common sources [3]. Our patient had a surgical intervention, which is one cause of the rise in the incidence of infections [3]. Back pain, fever, and neurological symptoms are only observed in 8%-15% of patients, and as their symptoms progress, they can present as cauda equina [3]. Four stages have been identified in spinal epidural abscess development: stage 1: back pain at the affected level of the spine, fever, and tenderness [3]. Radicular pain, nuchal rigidity, hyperreflexia, hypoasthesia, motor weakness, bowel and bladder incontinence, and paralysis [4,5]. The duration of each stage is unknown and can vary from hour to hour. Therefore, immediate MRI and treatment are recommended. 

## Conclusions

As the incidence of epidural abscesses is slightly increasing, it still requires a clinician to have high clinical suspicion due to improved prognosis and clinical outcomes. Epidural abscess has a very high mortality rate and noted progressive neurological deficits at presentation. Delay in care can cause permanent damage; therefore, if clinical suspicion is strong, the patient may still require urgent imaging. Concern for epidural abscesses should be acted upon immediately, as studies show that early surgical intervention improves outcomes. 
